# Carpal tunnel syndrome and the "double crush" hypothesis: a review and implications for chiropractic

**DOI:** 10.1186/1746-1340-16-2

**Published:** 2008-04-21

**Authors:** Brent S Russell

**Affiliations:** 1 Associate Professor, Division of Clinical Sciences, Life University, College of Chiropractic, 1269 Barclay Circle, Marietta, Georgia 30060, USA

## Abstract

Upton and McComas claimed that most patients with carpal tunnel syndrome not only have compressive lesions at the wrist, but also show evidence of damage to cervical nerve roots. This "double crush" hypothesis has gained some popularity among chiropractors because it seems to provide a rationale for adjusting the cervical spine in treating carpal tunnel syndrome. Here I examine use of the concept by chiropractors, summarize findings from the literature, and critique several studies aimed at supporting or refuting the hypothesis. Although the hypothesis also has been applied to nerve compressions other than those leading to carpal tunnel syndrome, this discussion mainly examines the original application – "double crush" involving both cervical spinal nerve roots and the carpal tunnel. I consider several categories: experiments to create double crush syndrome in animals, case reports, literature reviews, and alternatives to the original hypothesis. A significant percentage of patients with carpal tunnel syndrome also have neck pain or cervical nerve root compression, but the relationship has not been definitively explained. The original hypothesis remains controversial and is probably not valid, at least for sensory disturbances, in carpal tunnel syndrome. However, even if the original hypothesis is importantly flawed, evaluation of multiple sites still may be valuable. The chiropractic profession should develop theoretical models to relate cervical dysfunction to carpal tunnel syndrome, and might incorporate some alternatives to the original hypothesis. I intend this review as a starting point for practitioners, educators, and students wishing to advance chiropractic concepts in this area.

## Introduction

Upton and McComas [1] formalized the hypothesis of the "double crush" syndrome (DCS). They suggested that compression of an axon at one location makes it more sensitive to effects of compression in another location, because of impaired axoplasmic flow. Hypothetically, two lesions with little or no independent clinical ramifications, when combined, lead to appearance or magnification of symptoms [2]. Two areas of compression affecting the same axons do not, alone, meet the criteria of the hypothesis. By definition, a first lesion must render axons more susceptible to effects of a second, leading to more than just the combined, independent effects of two lesions [2]. Upton and McComas [1] used the double crush hypothesis to explain why patients with carpal tunnel syndrome (CTS) sometimes feel pain in the forearm, elbow, upper arm, shoulder, chest, and upper back. They also used it to explain failed attempts at surgical repairs when neither surgery nor CTS diagnosis appeared faulty. They claimed that most patients with CTS not only have compressive lesions at the wrist, but also show evidence of damage to cervical nerve roots.

The double crush concept has gained some popularity among chiropractors because it seems to provide a rationale for adjusting the cervical spine when treating CTS. An example of this emphasis on spinal care, implicitly grounded in the double crush hypothesis, can be found on the Web: "90% of all carpal tunnel patients are misdiagnosed. Only 10% of all carpal tunnel patients have the problem in their wrists. Most often the problem exists in the cervical spine with compression or irritation of the nerve root." [3]. Although the chiropractor making this claim may believe it, nothing in the scientific literature supports it. Other doctors of chiropractic accept that median nerve compression commonly occurs in the carpal tunnel but believe that neck problems also contribute to the syndrome. Although it seems that many of my chiropractic colleagues and students are unfamiliar with the term "double crush syndrome," nearly all share the conviction that the cervical spine is involved in CTS.

Are practicing chiropractors routinely using the double crush hypothesis? To learn more about this, and to precede and inform a more formal survey of scientific databases, I used the search engines Google and Ask to locate, "chiropractic double crush syndrome" and "chiropractic carpal tunnel syndrome." This was not meant to be an exhaustive search and I stopped after I had examined Web sites of 125 chiropractic practices promoting chiropractic care of the cervical spine for CTS. About half of these specifically referred to DCS or indirectly used the concept. Examples include:

"The first area I examine in a CTS case is not the wrist, but the neck. It is here that a group of nerves known as the brachial plexus comes out of the mid to lower neck region, then branches out to the arms, hands and fingers. If there is pressure on any of these nerves, especially the median nerve, the result may be CTS." [4]

"...nerve compression in the neck can block the flow of nutrients to the nerves in the wrist, making it more susceptible to injury. This is called the double crush syndrome." [5]

"...we have a comprehensive and unique six-point treatment plan for Carpal Tunnel Syndrome... [including] chiropractic adjustments of neck, shoulder, elbow and wrist.... CTS usually is due to pressure on the nerve at more than one location. (this is known as 'double crush [phenomenon].') " [6]

Some researchers have applied the double crush hypothesis to other nerve compression syndromes. Some implicate lesions in the elbow and thoracic outlet as contributing factors in CTS [7], and others have used the DCS hypothesis to explain nerve syndromes in other body areas (e.g., a chiropractic case report on DCS of the infrapatellar saphenous nerve [8]. However, I am concerned here with the original and most common application of the theory. Although chiropractors provide many different types of care to their patients, this discussion is oriented toward articular "adjustment", or manipulation – terms I will use interchangeably.

I have critiqued several studies with results thought to support or refute the hypothesis, including a chiropractic case report of DCS. Significant doubts exist about the validity of the original hypothesis, and many researchers since Upton and McComas have considered how cervical spine dysfunction might contribute to CTS. Several of these alternative ideas – many of which do not depend on nerve root compression – may be found toward the end of this article. I intend this as an overview of the double crush syndrome hypothesis that will spark further discussion of how CTS and chiropractic spinal care may be related.

## Methods

A PubMed search for "double crush carpal tunnel" yielded only 16 English language, peer-reviewed articles related to the cervical spine. A similar search of MANTIS yielded only one relevant article not retrieved by PubMed. I found many other related papers through cross reference and fortunate happenstance. I examined all that appeared relevant to the Upton and McComas definition of "double crush" but omitted from this review some papers that used the term incorrectly or inappropriately. I did not use many articles describing animal experiments because doing so would have gone beyond the scope of the paper, and other authors have reviewed this research.

For a separate set of searches for articles documenting chiropractic care of CTS, I used the terms "chiropractic carpal tunnel syndrome" in PubMed, "carpal tunnel syndrome" in MANTIS, limiting the search to chiropractic, and "carpal" AND "Tunnel" AND "syndrome" in the Index to Chiropractic Literature. These yielded 11 peer-reviewed articles and abstracts reporting chiropractic manipulative care of patients with CTS, only one of which was reported as a double crush syndrome.

## Discussion

### A DCS Overview: Upton and McComas' Theory

In articles in the late 1950s and early 1960s, Russell [e.g., [9]] suggested that "changes in the interstitial tissue of the nerves...may spread freely from one part to another, with the result that pathological changes in the nerve roots may, for example, influence vulnerability of the nerves at the wrist." [9]. Upton and McComas coined the term "double crush syndrome," and wrote: "...our hypothesis is that neural function is impaired because single axons, having been compressed in one region, become especially susceptible to damage at another site." [1] They developed their theory following study of 115 patients who had been determined (through electrophysiological examination) to have an entrapment neuropathy at either the elbow or the carpal tunnel. Eighty-one of these patients also had cervical spondylosis, complaints of neck pain and stiffness, history of neck injury, symptoms of dermatomal sensory abnormalities, or electromyographic signs of denervation apparently related to cervical nerve roots. However, in most of these cases, there was no evidence that the cervical pathology (e.g., spondylosis, stiffness, history of injury) actually affected the nerve roots. Upton and McComas suggested that there could be a relationship between the symptoms in the wrist and the neck but did not demonstrate it.

### What's Wrong with the Double Crush Hypothesis?

Upton and McComas based their hypothesis on an interference with axoplasmic flow (more commonly known as axonal transport), the mechanism by which trophic substances manufactured in a neuronal cell body (e.g., proteins, lipids, neurotransmitters) are carried away along the peripheral processes of the neuron (anterograde transport) and products of lysosomal breakdown are transported back to the cell body (retrograde transport) [10]. If an axon is sufficiently compressed or severed, it is detached from its source of nutrients, and its distal portion undergoes degeneration [10]. To address a point of possible confusion, the peripheral process of a sensory neuron actually is an elongated dendrite. However, it is called an axon in common usage [11] and has properties similar to those of a peripheral axon, including the functions of axonal transport.

**Figure 1:**
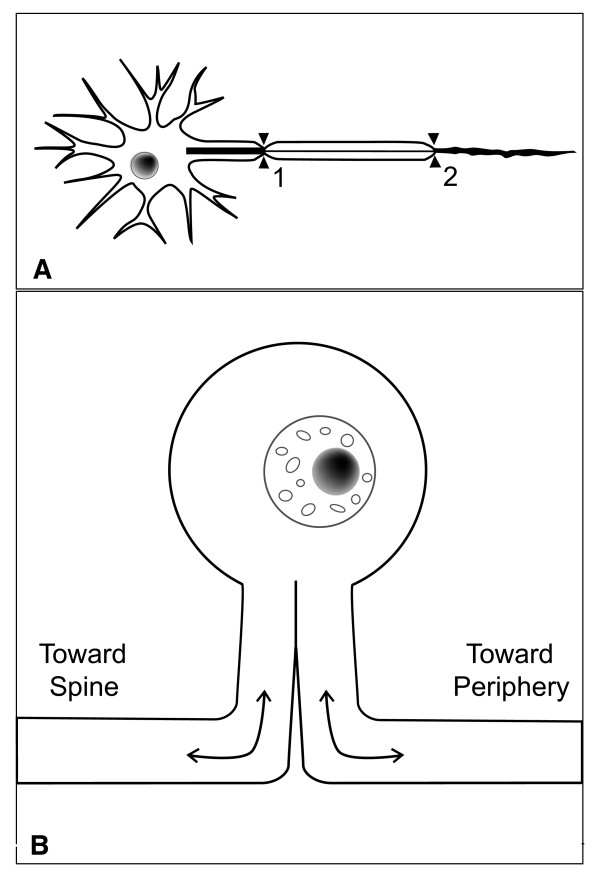
**A:** graphic representation of motor neuron, with two sites of compression along single axon. Proximal compression affects intraneural circulation and impulse transmission, with second compression more than doubling those effects. **B:** Graphic depiction of cell body of a sensory neuron. Axonal transport to and from the periphery is mechanistically separate from transport to and from the spinal cord. Compression of the proximal (left) branch is unlikely to affect transport for the distal (right) branch.

Double crush syndrome would have to involve direct axonal continuity from the proximal to the distal lesion sites [12,13]. For example, motor aspects of carpal tunnel syndrome (e.g., muscle weakness) could qualify, because cell bodies of spinal motor neurons are found in the anterior horn of the spinal cord; proximal compression of axons in an anterior (ventral) cervical nerve root and of the same axons in the median nerve at the carpal tunnel would constitute two sites of compression along the same neural processes (Figure 1A). Although the hypothesis could be applied in the case of any dual compression of the same axons, most literature has implicated nerve roots as the site of proximal compression.

Direct axonal continuity between spinal roots and peripheral lesions does not occur with sensory neurons, however. At each spinal level, cell bodies for sensory neurons are in ganglia near the distal (lateral) end of their posterior (dorsal) root, outside the spinal column. Thus, cell bodies lie between their peripheral processes (laterally) and most of their posterior roots (medially) [14]. Anterograde transport moves materials from the cell body (laterally into the peripheral branch; and medially into the nerve root). These distal and proximal neuronal processes have separate sets of microtubules, for separate axonal transport systems [15] (Figure 1B). Wilbourn and Gilliatt [12] and Morgan and Wilbourn [13] asserted that compression of the posterior nerve root would not affect axonal transport to the periphery, and that it is therefore not appropriate to use the DCS hypothesis to relate sensory disturbances of CTS (e.g., pain, numbness, tingling) to compression of cervical nerve roots – but this is how the concept has most often been used.

Axoplasmic flow was not well understood at the time Upton and McComas proposed DCS, and failure of that particular explanation would not necessarily render invalid the entire concept, nor would it rule out the impact of compression at sites other than the roots. Perhaps it is significant that action potentials generated at distal sensory nerve endings bypass the cell body (unlike axonal transport) as they are transmitted along the peripheral branch to the central branch [16]. Other possible explanations discussed by Osterman [17] include: interruption of lymphatic or venous drainage at the proximal site making the distal nerve more susceptible to entrapment [18]; or proximal endoneurial edema affecting intraneural vascular circulation distally [19]. I consider other alternatives further on.

### DCS: Basic Science Experiments and Conclusions of other Reviewers

Several researchers have attempted to artificially produce double crush syndromes in animals. Although it is beyond my scope to discuss them all, interested readers should seek out reviews by Wilbourn and Gilliatt [12] and Swenson [2] and a book chapter by Cheng [20].

It seems logical that two sites of compression affect axonal transport, impulse conduction speed, and action potential amplitude more than a single lesion, and that greater compression would have more effect than slight amounts. Experiments with animals leave little doubt that these presumptions are true [19,21-23].

Nemoto, et al. [21] compared effects of two clamps with those of one on sciatic nerves of dogs. Although a single, mild compression produced a partial conduction block and mild axonal degeneration, mild compression at two sites produced a complete conduction block and severe degeneration in some animals. They also found that recovery was poor if only one of two clamps were removed, supporting Upton and McComas' concern that carpal tunnel release may fail to relieve patient symptoms when other sites of compression are present. Dellon and Mackinnon [22] also tested effects of dual compression, using Silastic bands just proximal and just distal to the bifurcation of sciatic nerves in rats. Circumferences of bands were adjusted under a microscope and produced no visible nerve compression. Even so, a band at a single site caused a decrease in action potential amplitudes and conduction velocity, and an additional site caused significantly greater decrease in both. Dellon and Mackinnon [22] did not provide exact numbers but, judging from graphs provided, effects of two sites of compression apparently were less than double the effects of one site – a finding that would not support the original DCS hypothesis.

Cheng [20] stated that, "animal studies rather convincingly support the 'double crush' phenomenon." On the other hand, in their reviews, Swenson [2] and Wilbourn and Gilliatt [12] criticized experimental designs and statistical analyses of the above and remained unconvinced. Both commented that a number of studies have documented that two or more lesions have a greater effect than one, but that no research had conclusively shown anything greater than an additive effect – not the magnifying effect that had been theorized.

### Overview of DCS Literature on Human Patients

A number of clinical studies of the double crush hypothesis have documented patients with CTS who also had cervical spinal problems. Hurst et al. [24] is a retrospective review of the medical records of 888 patients (1,000 wrists) who underwent carpal tunnel release between 1950 and 1979. They noted that 11% (95 patients) had cervical arthritis and 41% of these had bilateral CTS. Although the authors suggested that their findings might support the double crush theory, they did not demonstrate actual compression on cervical nerves. Eason et al. [25] retrospectively reviewed records of 34 patients (47 wrists) who had suboptimal results following carpal tunnel release. Twenty-five of these (38 wrists, 81%) had "symptoms and/or signs of cervical spine disease," including neck pain; shoulder, arm, elbow, or forearm pain; decreased cervical range of motion; unilateral decreased biceps reflex; and previous neck injury or surgery. Seventeen patients with neck pain had abnormal findings on x-rays, including cervical disc space narrowing and osteophytes, but electrodiagnostic findings were reported only for the wrist. Therefore, whether any patients actually had cervical radiculopathy remains unknown. Baba et al. [26] reviewed records of 483 patients who underwent cervical cord and nerve root decompression and upper arm peripheral nerve decompression (177 at the carpal tunnel, 108 at the cubital tunnel). Of these, 65 with both cervical and peripheral signs and symptoms were considered to have double-crush lesions. The authors recognized the potentially greater diagnostic challenge posed by multiple simultaneous lesions. They also recognized the importance of deciding which area to treat first, noting that remedy directed at only a single area may not resolve a patient's complaints and that delay could result in nerve damage.

All of these researchers claimed findings supportive of the double crush hypothesis, and they have been cited by other researchers and chiropractors as evidence of validity of the syndrome. However, in each case, although authors documented patients with both cervical problems and CTS, they did not demonstrate that the two were clinically tied.

Carpal tunnel syndrome has been estimated to affect approximately 3%–4% of the general population [27,28] and it is more common among women aged 40 years or older. The incidence of clinically recognized cervical radiculopathy has not been extensively studied but apparently is found in less than one-half of 1% of the general population, with a peak at the age range of 50–54 [29-31]. Surely for a patient to have both conditions at once, without a clinical relationship, would be infrequent. Golovchinsky [32] investigated such simultaneous occurrence, using a chi square analysis of 327 patients, and found that the two conditions occurred together more often than would be likely through chance alone. He concluded that double crush syndrome exists as a separate clinical entity [33]. However, he also acknowledged that, "The exact neurophysiological and cellular mechanisms of this phenomenon...are not clearly established or universally accepted." [33] Morgan and Wilbourn [13] reviewed nerve conduction and electromyographic findings of 10,743 hands that had been diagnosed with CTS; only 0.03% (three patients) satisfied their stringent anatomical and pathophysiological criteria for DCS.

Richardson et al. [34] analyzed cases of C6, C7, and C8 cervical radiculopathy and "exploited" the fact that median nerve sensory fibers ordinarily use the C6 and C7 roots and the motor fibers primarily use C8 and T1. Investigators hypothesized that abnormal sensory conduction of the median nerve would be found more frequently in those patients with C6 or C7 radiculopathy, and that abnormal motor conduction would be detected more often with C8 radiculopathy. Their findings did not support their hypotheses, however; while a relatively high number (22.1%) of cervical radiculopathy patients had abnormalities of the median nerve, cervical nerve levels frequently did not correlate as expected. The authors concluded that current understanding of peripheral nerve anatomy and physiology was inconsistent with the double crush theory of CTS. More recently, using a similar premise, Kwon et al. [35] also failed to find a significant correlation.

In another recent study, although Flak et al. [36] examined 30 patients with both carpal tunnel syndrome and cervical radiculopathy (using x-ray, magnetic resonance imaging, electroneurography, and somatosensory evoked potentials (SSEP), and asserted that DCS does exist, I was left with more questions than answers. For example, symptoms of CTS and cervical radiculopathy can be very similar and the authors did not elaborate in the inclusion criteria on how they were differentiated. Also confusing is that inclusion depended on electrodiagnostic confirmation of CTS but they described abnormalities in only 22 patients. They reported a statistical correlation between median nerve and brachial plexus, for both decreases in conduction amplitudes and increases in conduction latency, but did not report a correlation coefficient. They found "compliance" of lateralization of intervertebral foramen narrowing and median nerve SSEP abnormality in "71.4%" (21.4 patients? 42.8 sides?), but did not supply figures for left, right, or bilateral occurrence of either condition. They also did not provide results of Wilcoxon, Ancova, Anova, Chi Square, and multiple regression analyses, described earlier under "methods."

### Chiropractic Literature on DCS

There are relatively few peer-reviewed articles [37-47] on chiropractic care of CTS (Table 1), and only Mariano et al. [38] claimed a patient to have had a case of DCS. Diagnosis of cervical radiculopathy was based on pain in the neck and upper back, pain radiating into the left arm, numbness and paresthesia of the left hand, and palpatory tenderness, muscle spasm, and facet joint hypomobility of the cervical spine [38]. Other findings were normal, except for slight weakness of the left abductor pollicus brevis muscle. X-rays revealed disc degeneration and stenosis of the intervertebral foramina at the levels of C4–7. Additional diagnosis of CTS was based upon electromyographic findings. The patient was treated with spinal manipulation, therapeutic ultrasound, electrical nerve stimulation, and a home traction unit, all directed to the neck and upper back. The CTS was treated with a wrist splint.

**Table 1:** Peer-reviewed articles reporting chiropractic care of carpal tunnel syndrome.

**Author(s)**	**Year**	**Methods of care**
Ferezy and Norlin [37]	1989	spinal & extremity manipulation, soft tissue massage, cervical pillow
Mariano et al. [38]	1991	spinal manipulation, therapeutic ultrasound, electrical nerve stimulation, home traction unit (cervical), wrist splint
Bonebrake et al. [39]	1993	spinal and extremity manipulation, soft tissue manipulation and massage techniques, intersegmental traction, microwave diathermy, ultrasound, dietary modifications, mineral supplements
Valente and Gibson [40]	1994	spinal and extremity adjustments
Petruska [41]	1997	extremity manipulation (machine-assisted axial wrist traction), microcurrent, nutritional supplementation, rehabilitative exercises
Manello et al. [42]	1998	spinal and extremity manipulation, soft tissue manipulation, exercises
Dunphy et al. [43]	1998	extremity manipulation (pneumatic traction device)
Davis et al. [44]	1998	(randomized clinical trial) Chiropractic group: spinal and extremity adjustments, myofascial massage, ultrasound, wrist splint Medical group: oral ibuprofen, wrist splint
Perez de Leon and Auyong [45]	2002	extremity manipulation, flexion-distraction, ultrasound, cryotherapy, muscle stimulation, deep tissue massage, wrist supports, vitamin/mineral supplements, exercise
Brunarski et al. [46]	2004	extremity manipulation (machine-assisted axial wrist traction)
George et al. [47]	2006	myofascial therapy (Active Release Technique)

The Mariano [38] report leaves some doubt whether the patient actually had CTS. The Phalen and Tinel tests were negative, though these maneuvers (especially the Tinel test [48]) do have a significant false negative rate. Secondly, diagnosis of CTS was made on the basis of EMG findings, whereas sensory and motor conduction velocity are the more commonly used electrodiagnostic measures for CTS [49]; Mariano et al. [38] did not report whether nerve conduction velocity was evaluated. If we consider the doubts of Wilbourn and Giliatt [12] and Morgan and Wilbourn [13], we could probably accept this as a case of DCS only if (1) we assume that Mariano's patient's symptoms originated at the cervical spine (not the wrist), and (2) we accept that this patient had a form of CTS involving only motor fibers (as manifested by abductor pollicus weakness and EMG findings). Another possibility is that the Mariano case actually was cervical radiculopathy, alone.

### Alternatives to the Upton and McComas' Model

Some have suggested that carpal tunnel syndrome is a problem of the upper body rather than of the wrist, per se. According to Donaldson et al. [50], "Explanations for CTS have often focused narrowly on the pathophysiology [of] nerve disturbance in the extremity without... a wider integration of physiological systems in the etiology and maintenance of CTS." [50] Although no single theory has displaced the Upton and McComas model, alternatives are worth exploring.

Murray-Leslie and Wright [51] found greater degrees of intervertebral disc narrowing and lateral epicondylitis in a group of CTS patients than in a control group. They speculated that there might be connective tissue changes that allowed soft tissue degeneration at these sites. Osterman [17] interpreted this conjecture as "a possible underlying generalized condition of connective tissue". Similarly, Shimpo [52] suggested that the clinical association that Upton and McComas observed was caused by the coexistence of osteoarthritis affecting the cervical spine and the limb joints, with independent nerve lesions at each level.

Others have suggested that compression of the median nerve in the carpal tunnel is simply the most evident pathological feature of a multi-site problem resulting from mechanically stressful body postures and physical activities. Richardson et al. [34] proposed that, "Upper extremity weakness and pain in patients with [cervical radiculopathy] may cause changes in biomechanics and usage patterns leading to increased upper extremity edema with resultant increased carpal tunnel pressures." Bednarik et al. [53] suggested that either (1) common extrinsic factors involving mechanical stress to both the cervical spine and upper extremities might simultaneously lead to accelerated spondylosis and entrapment syndromes, or that (2) weakness or poor coordination caused by cervical myelopathy might lead to compensatory overuse of the hand. Leahy [54] doubted the role of the carpal tunnel itself and implicated a number of sites where nerves may become entrapped in the shoulder, arm, and forearm. He mainly focused on locations where nerves are known to pass under or through muscles, because factors such as muscle spasm, adhesions, or edema can cause nerve compression at such sites [55,56].

The above ideas are represented, or at least implied, by the more elaborate model offered by Novak and Mackinnon [57], summarized as follows:

1. Certain postures or positions will increase tension or increase pressure at sites where nerves are entrapped. Placing a nerve under tension – e.g., the median nerve with wrist extension and the brachial plexus with arm elevation – may compromise neural blood supply. Pressure on a nerve at an entrapment site may cause increased neural edema, inflammation, fibrosis, and decreased neural mobility.

2. If a posture places a muscle in shortened position, it will, over time, undergo adaptive shortening. When stretched, the shortened muscle may produce local discomfort, and if the muscle crosses over a nerve, the nerve may become secondarily compressed.

3. Abnormal postures will cause some muscles to be elongated or shortened (versus optimal musculoskeletal alignment). The muscles will undergo anatomic, biomechanical, and physiologic changes, resulting in muscle weakness. With weakness in some muscles, others will be recruited to compensate, and the cycle of muscle imbalance will continue.

Donaldson et al. [50] and Skubick et al. [58] proposed a mechanism through which asymmetrical function of neck muscles could cause carpal tunnel syndrome: excessive afferent input from an injured or dysfunctional neck muscle blocks normal inhibition at the gamma motoneuron level, leading to inappropriate coactivation with other muscles (the forearm flexors, in the case of CTS) during movement. Donaldson [50] reported having observed abnormal EMG activity of flexor and extensor muscles of the forearm concurrent with head rotation in CTS patients. Forearm flexor tendons, which pass through the carpal tunnel, are pulled across the transverse carpal ligament in flexion and anterior surfaces of carpal bones in extension, somewhat like a belt across a pulley. Excessive forearm muscle activity increases the load borne by the tendons, and therefore mechanical stress, which may lead to tenosynovitis. Although this proposal may be original, tenosynovitis has been widely cited as a part of CTS pathology [59-61]. It is believed that enlargement of tendon sheaths increases the volume of contents of the carpal tunnel, which increases the internal pressure, leading to nerve compression.

In a case series of 18 CTS patients and using surface EMG, Skubick et al. [58] found asymmetrical activity in the sternocleidomastoid, cervical paraspinal, forearm flexor, and forearm extensor muscles. Specific neuromuscular retraining – simple neck exercises – resulted in improved sternocleidomastoid symmetry, decreased forearm flexor activity, and improvement in nerve conduction measures for every patient. By the end of treatment, 10 patients reported that they were symptom-free.

Among patients with diabetes and CTS, some have suggested that diabetes could be considered the first "crush" [17,25,53,62]. The rationale seems to be a statement by Upton and McComas that, "This hypothesis does not exclude the development of entrapment syndromes in patients with a generalized subclinical neuropathy." [1] It does appear that axonal transport is altered in diabetes [63]. A greater than expected incidence of CTS has been documented in diabetic [64], obese [65,66], and chronic kidney disease [67] patients. However, Upton and McComas acknowledged that most patients with multiple entrapment neuropathies had no evidence of these or other clinical factors [1]. Carrying this concept even further, Nathan et al. [68] found that, among workers with CTS, there was a 19% greater lifetime use of tobacco, 75% greater history of alcohol abuse, and a 5% greater use of caffeine. All doctors caring for patients with carpal tunnel syndrome should be aware of these factors. In regards to DCS, though, equating these conditions with actual compressive lesions seems a bit like "comparing apples to oranges" and a liberal interpretation of the original concept.

### Chiropractic Considerations

Any chiropractor who wishes to use the double crush hypothesis as justification for a cervical adjustment approach to CTS should be aware that the hypothesis is controversial. Although specific diagnostic criteria exist for carpal tunnel syndrome, reality of the double crush syndrome has not been established. Although DCS is a relatively obscure topic in the spectrum of health care, it is an important area for chiropractic and one in which practitioners should exercise caution in both treatment choices and public statements.

Before considering cervical adjustment a realistic option for CTS patients, we must ask, "How does it work?" Neither the original DCS hypothesis nor other proposed models directly support the role of spinal manipulation, although the Novak and Mackinnon [57] or Donaldson et al. [50] and Skubick et al. [58] hypotheses may be good starting points for development of a new model. The scenario described by Novak and Mackinnon [57] dovetails neatly with the kinetic chain interactions that many chiropractors have used to explain relationships between remote regions of the spine or between spinal and extremity lesions. It also allows a rationale for the use of spinal and extremity manipulation, myofascial therapy, therapeutic exercises, and other treatments.

Professional and personal experiences have led me to conclude that chiropractors' anecdotes probably reflect reality: some percentage of patients with carpal tunnel syndrome probably do improve with cervical spine manipulation. Unfortunately, limited research on chiropractic and CTS has not given us sufficient evidence to make that claim, more formally. Perhaps interested practitioners should begin by documenting effectiveness, success/failure rates, or costs of chiropractic care for CTS. Once efficacy has been scientifically established, it would be appropriate to explain how cervical adjustment leads to relief from peripheral dysfunction. Members of our profession have been accused, in general, of making unsubstantiated claims to treatment efficacy – so far, chiropractic faith in the double crush hypothesis appears to be an example.

## Conclusion

If the original double crush mechanism is involved at all in the clinical development of carpal tunnel syndrome, it is probably rare and likely not related to sensory disturbances. Some alternative models might better explain a relationship between cervical spine dysfunction and CTS, and would better support the use of spinal (and extremity) manipulation for CTS. Many DCS researchers have made cautionary statements along these lines: some patients may suffer from more than one lesion at a time, all should be examined at multiple sites for compression, and treatment should be directed at all affected sites. This line of reasoning might allow a greater role for chiropractic as a conservative approach – surely a reasonable alternative to multiple surgeries. The profession should further develop theoretical models to relate cervical dysfunction to CTS, produce better documentation of chiropractic as effective care for CTS, and demonstrate that chiropractic makes sense economically. In short, we have work to do.

## Abbreviations

CTS: carpal tunnel syndrome; DC: Doctor of Chiropractic; DCS: double crush syndrome; EMG: electromyography, electromyographic; SSEP: somatosensory evoked potentials.

## Competing interests

The author declares that he has no competing interests.
